# Development of a patient-reported outcome measure (PROM) and change measure for use in early recovery following hip or knee replacement

**DOI:** 10.1186/s41687-020-00262-1

**Published:** 2020-11-07

**Authors:** Louise H. Strickland, David W. Murray, Hemant G. Pandit, Crispin Jenkinson

**Affiliations:** 1 Oxford Orthopaedic Engineering Centre (OOEC), Nuffield Department of Orthopaedics, Rheumatology and Musculoskeletal Sciences (NDORMS), University of Oxford, Botnar Research Centre, Windmill Road, Headington, Oxford, OX37LD England; 2 Leeds Institute of Rheumatic and Musculoskeletal Medicine (LIRMM), University of Leeds, Chapel Allerton Hospital, Leeds, LS7 4SA England; 3 Nuffield Department of Population Health, University of Oxford, Headington, Oxford, OX37LF England

**Keywords:** Early recovery, Postoperative, Knee arthroplasty, Hip arthroplasty, Patient-reported outcome measure, Questionnaire, Food and Drug Administration (FDA), Validity, Reliability

## Abstract

**Background:**

Hip and knee replacement are effective procedures for end-stage arthritis that has not responded to medical management. However, until now, there have been no validated, patient-reported tools to measure early recovery in this growing patient population. The process of development and psychometric evaluation of the Oxford Arthroplasty Early Recovery Score (OARS), a 14-item patient-reported outcome measure (PROM) measuring health status, and the Oxford Arthroplasty Early Change Score (OACS) a 14-item measure to assess change during the first 6 weeks following surgery is reported.

**Patients and methods:**

A five-phased, best practice, iterative approach was used. From a literature based starting point, qualitative interviews with orthopaedic healthcare professionals, were then performed ascertaining if and how clinicians would use such a PROM and change measure. Analysis of in-depth patient-interviews in phase one identified important patient-reported factors in early recovery which were used to provide questionnaire themes. In Phase two, candidate items from Phase One interviews were generated and pilot questionnaires developed and tested. Exploratory factor analysis with item reduction and final testing of the questionnaires was performed in phase three. Phase Four involved validation testing.

**Results:**

Qualitative interviews (*n* = 22) with orthopaedic healthcare professionals, helped determine views of potential users, and guide structure. In Phase One, factors from patient interviews (*n* = 30) were used to find questionnaire themes and generate items. Pilot questionnaires were developed and tested in Phase Two. Items were refined in the context of cognitive debrief interviews (*n* = 34) for potential inclusion in the final tools. Final testing of questionnaire properties with item reduction (*n* = 168) was carried out in phase three. Validation of the OARS and OACS was performed in phase four. Both measures were administered to consecutive patients (*n* = 155) in an independent cohort. Validity and reliability were assessed. Psychometric testing showed positive results, in terms of internal consistency and sensitivity to change, content validity and relevance to patients and clinicians. In addition, these measures have been found to be acceptable to patients throughout early recovery with validation across the 6 week period.

**Conclusions:**

These brief, easy-to-use tools could be of great use in assessing recovery pathways and interventions in arthroplasty surgery.

## Background

The incidence of arthritis is increasing [1]. The World Health Organisation (WHO) has identified arthritis as one of the top ten disabling conditions. As the number of people experiencing arthritis increases, thus the number of patients requiring surgical intervention increases. It is estimated that approximately 200,000 hip and knee replacements are performed in the United Kingdom (UK) annually [2], with the number being 1 million in the United States (US) [3, 4]. This number is anticipated to continue to grow significantly over the next 10 years [5]. Despite increases in the frequency of this procedure, the way we measure recovery has not changed in recent years.

Optimising perioperative recovery is critical to enhance patient care, ensure timely discharge from the patient, clinician and hospital perspective and improve short and long-term outcomes after surgery [6]. However, until now, there has been debate about how to measure recovery with previously used measures in early recovery not patient-reported.

Prior to commencing this study, a systematic literature review was performed to evaluate the need for an early recovery PROM or change measure in this patient population [7]. The most important finding from this review is that whilst 15 instruments were identified to assess postoperative recovery, none were found to fulfil all quality criteria [8] and be valid for assessing early postoperative recovery in the hip or knee arthroplasty population. This specifically revealed that previously used measures were found to be inappropriate to accurately evaluate the quality of recovery and lacked precision. Only seven out of the 15 instruments included any orthopaedic patients in their development. Within those seven, less than 15% of those patients were orthopaedic. Thus limiting the applicability of these instruments as it is likely that recovery factors important to patients undergoing orthopaedic surgery are significantly different to recovery factors in patients undergoing other types of surgery. Being able to measure patient-reported outcomes following arthroplasty could be of great benefit in clinical trials involving medication, care pathways and implant selection. It could also potentially work to optimise routine care by allowing provision of appropriate, safe, timely care and interventions.

The process of development for a PROM and a measure to determine postoperative change since surgery was therefore begun with initial qualitative work being performed to facilitate concept understanding and item generation. The Food and Drug Administration (FDA) guidelines [9] provide a thorough outline by which new patient-reported outcome measures (PROMs) should be developed. Item generation comes directly from patient statements and from the patient population the tools are being designed to serve. Throughout the entire process including item generation, selection of candidate items, wording changes and item reduction, a detailed item tracking matrix was maintained. The item tracking matrix provides ease of identification in item modifications, direct patient sources, and a record of item deletions.

## Methods and phases

**Fig. 1:**
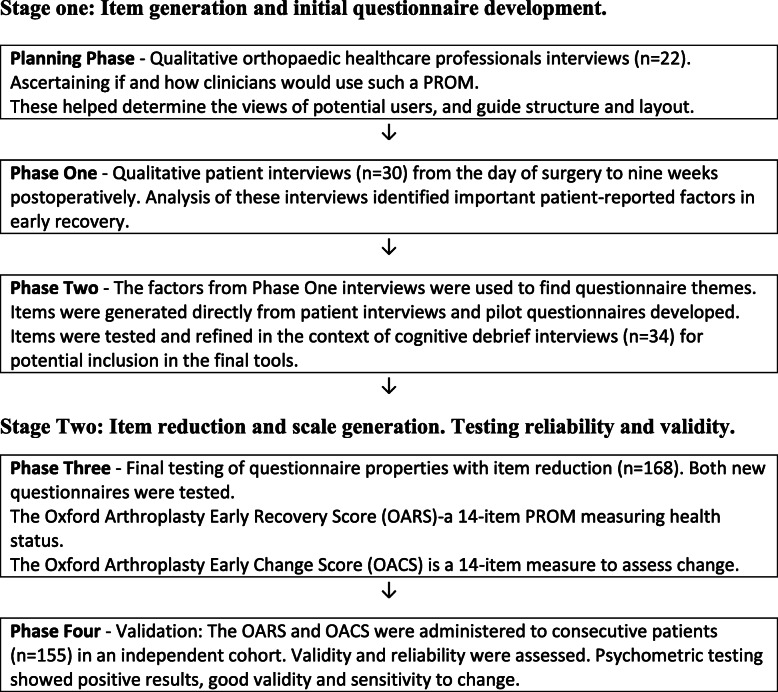
Study stages and phases

The Oxford Arthroplasty Early Recovery Score (OARS) and the Oxford Arthroplasty Early Change Score (OACS) were developed and tested through mixed methods research study and was carried out across two stages (five phases) in strict accordance the Food and Drug Administration (FDA) guide [9] for best practice in PROM development (Fig. 1).

### Stage one: item generation and initial questionnaire development

#### Planning phase

The initial planning phase, consisted of exploratory semi-structured interviews (*n* = 22) to explore orthopaedic healthcare professionals’ experience and perspective of early recovery for patients undergoing total hip arthroplasty (THA) or total knee arthroplasty (TKA). These were used to guide structure and layout of the questionnaires.

#### Design

In the planning phase interviews, semi-structured interviews were utilised to explore the experience and perspective of the early recovery period by healthcare providers caring for patients undergoing THA or TKA. These interviews, were guided by a list of interview prompts, which facilitated further exploration of areas of the topic that need to be covered by the interviewer [10]. The interview guides were standardised and consisted of open-ended questions and prompts. These were developed by the research team and patient partners.

#### Analysis

An in-depth pragmatic thematic analysis method [11] was utilised. Thematic analysis facilitates identification of themes or commonalities in interview transcripts. It helps organise and understand data. In this research developing a patient-reported recovery measure, it is important to fully explore and understand the themes that are of importance in postoperative arthroplasty recovery for patients and healthcare providers. These interviews provided background and clinician perspective to the possible new PROM.

#### Phase one

Phase one of the study, patients undergoing THA or TKA were interviewed (*n* = 30) during the early perioperative period between the day of surgery and discharge from the surgeons care between 6 and 8 weeks. A conceptual model was utilised when developing the new tools [12]. In addition, results from the phase one qualitative findings were considered for making decisions about item reduction.

#### Design

Phase one interviews, consisted of semi-structured interviews to explore the experience and perspective of patients during the early recovery period undergoing THA or TKA. These interviews, were guided by a list of interview prompts, which facilitated further exploration of areas of the topic that need to be covered by the interviewer [10]. The interview guides were standardised and consisted of open-ended questions and prompts. These were developed by the research team and patient partners.

#### Analysis

As in the planning phase, an in-depth pragmatic thematic analysis method [11] was utilised. Thematic analysis facilitates identification of themes or commonalities in interview transcripts. It helps organise and understand data. Coding is the technique by which themes are identified and organised. This method of analysis was chosen for several reasons. It was vital for this research developing a patient-reported recovery measure to fully explore and understand the themes that are of importance in postoperative arthroplasty recovery for patients and healthcare providers. As the tool was being designed for use in both clinical and home settings (the latter after discharge), the tool needed to be meaningful and effective in a real world setting [13]. Following immediate exact word-for-word transcription, interviews were anonymised to remove any participant identifiable data.

These transcribed interviews were then imported into NVivo software (NVivo qualitative data analysis Software; QSR International Pty Ltd. Version 11, 2015) and analysis performed. Themes that were important to patients undergoing hip or knee replacement were recorded. This process is known as coding in qualitative research [14]. Initial coding of the interviews was performed independently by two reviewers, the researcher and an expert colleague in qualitative research, to ensure thorough coverage of the work. Interviews were coded based on the patients’ words and context. The interviews were analysed in an iterative ongoing basis. This technique is designed to elucidate any new themes that may emerge as the study is being performed and allows for the iterative process of interview adaptation to occur. If any new areas come to light during the earlier interviews, they can be added into subsequent interviews as interview prompts. This too helps to ensure full coverage of the concept being explored [13]. The sample size for participants was guided by data saturation [15, 16] which is the time at which subsequent interviews did not produce any new themes. Interviews were coded based on the participants’ words and context. This important part of the analysis was performed independently by two researchers and discussed. Any unresolved concerns were taken to a third researcher for further resolution.

Item generation came directly from patient statements and from the patient population the tools are being designed to serve. Following thematic analysis and coding of the qualitative interviews, a list of potential sample items were created for each theme. These statements included items from all patient interviews and all themes from the Phase One analyses. These were reviewed by the expert and patient panel which included two surgeons, two nurses, an anaesthetist, two hip and knee arthroplasty patients, a psychometrician with a particular interest in patient related outcomes, and one patient caregiver. The purpose of this panel was to review and evaluate potential items. In addition, ideas and suggestions for layout and response options were also discussed.

#### Phase two

The first iteration of the OARS contained 18 items. The items in this PROM covered all aspects of the early recovery period. The first iteration of the OACS contained 25 items. This change measure included items designed to cover the concepts of early recovery and the change that may occur during the first 6 weeks. The items covered all themes reported by patients.

#### Design

The candidate questionnaires were tested once during the patients’ hospital stay and cognitive debrief interviews (*n* = 34) were used to assess items for face and content validity. Validity of an outcome measure is the extent to which it measures what it claims to measure. This is assessed through consideration and evaluation of several different aspects, including content and construct validity [17]. Changes were made accordingly to the questionnaire.

#### Analysis

Patients were requested to complete the questionnaire in the context of cognitive debriefing. These techniques allow the interviewer to determine the meaning a participant gives to questions and why they selected particular response options [9, 18]. All participants received both the OARS and OACS at the time of testing. These draft questionnaires were administered once to 34 patients in the early postoperative period during hospitalisation (days 0 to 8). The participants were then asked to discuss the items, the reason for their answers and the meaning they attributed to them. During these interviews, participants were asked to discuss how thorough they felt coverage was of the topic of recovery after TKA or THA. In addition, they were asked if the questions were easily understandable and if they were relevant to their particular situation. These interviews were audio recorded and transcribed verbatim. This led to the first version of test questionnaires being developed that were refined in the following phases.

Prior to testing, a translatability assessment (TA) was performed on the two new measures. TA has been recognised as an important part of the questionnaire development process [9]. It provides insight into what extent the items in the questionnaires can be translated into other languages [19]. This is of particular importance for use in cross cultural trials. Changes may be made to the wording of some items as a consequence of this procedure. In addition, a concept elaboration document (CED) was created to fully define and clarify question items and the meanings attributed to them. This was developed in combination with the author [20] and specialist translators, to provide specific detail regarding the explicit line-by-line meaning of items and concepts, providing clarification of each line of the questionnaire [21].

### Stage two: item reduction and scale generation. Testing reliability and validity

#### Phase three

Final questionnaire development and testing was performed in phase three.

#### Design

Patients (*n* = 168) were given questionnaires on days 1,2,3,7,14 and 6 weeks following either hip or knee replacement surgery. They were administered on days 1, 2, 3, 7 and 14 in the early postoperative period and also at 6 weeks following surgery. Exploratory factor analysis (EFA) was used to explore the dataset and determine what latent underlying constructs are being measured [22]. EFA evaluates the scale properties and aids in removing non-response level or are not internally consistent [23, 24]. All of the items for the OARS and OACS were put into a factor analytic model (Varimax with Kaiser Rotation). Varimax rotation was selected as it facilitates data pattern interpretation. EFA was performed on the most populous time point (day 1 testing). Only factors which gained an eigenvalue of > 1 were retained. Selection and decision making on the number of factors to be retained can be determined by multiple means of testing including eigenvalues and factor loadings [25]. In participants who were discharged prior to day three, questionnaires with stamped self-addressed envelopes were provided. The specific testing time points were chosen to maximise the information acquired from participants and also to provide thorough coverage of the early recovery period.

#### Analysis

In phase three, both exploratory factor analysis and item reduction were performed using SPSS 25 software. Kaiser-Meyer-Olkin Measure of Sampling Adequacy (KMO) and Bartlett’s Test of Sphericity tests were performed to determine if the data was appropriate for factor analysis. Frequency tables were created and examined for floor and ceiling effects. An initial principal component analysis (PCA) was undertaken to determine if any of the items were not suitable for analysis or outliers. This process sorts variables into factors and indexes the amount of variance from each. This number is called the eigenvalue and, as previously mentioned, values above one are considered statistically significant and meaningful. Descriptive statistics and frequency tables reported. Exploratory factor analysis (EFA) was carried out on the most populous time point to determine what constructs underlie the data and to determine redundancy in any of the items. PCA with Varimax rotation was performed on days 1, 7, 14 and at 6 weeks. PCA is utilised to reduce the data into a smaller number of components. Varimax rotation was selected as it facilitates data pattern interpretation. Item reduction was reported. In this group, those with stronger factor correlations (above 0.5) are considered to have loaded on those factors [26]. The weaker, or non-loading, items can then be considered for removal. Internal consistency was also reported using the alpha statistic, known as Cronbach’s alpha, indicates the extent to which there is a pattern of responses to items. It is a commonly used statistical test for this purpose [27], with scores above 0.7 considered acceptable.

#### Phase four

Validation of questionnaires responsiveness and sensitivity to change were measured in phase four. These are important, closely related qualities in validating outcome measures, particularly if they are potentially being used in clinical decision making and trials [28]. The results from the OARS and OACS were evaluated for responsiveness, a measure’s ability to detect clinically important changes and sensitivity to change over time, a statistical feature of a measure. The initial testing time point was measured and compared with the means of additional testing points through 6 weeks. Construct validity is the extent to which a questionnaire measures what it claims to measure [29]. Comparison and correlation of the previously hypothesised dimensions of the SF-36v2 were made in relation to the new OARS to assess construct validity [29].

#### Design

The two final questionnaire versions were distributed to consecutive patients (*n* = 155) in a cohort of hip and knee replacement patients. They were again administered on days 1, 2, 3, 7 and 14 and also at 6 weeks following surgery. In addition a widely used, validated, generic health measure the Short Form-36 version 2 Acute (SF-36v2), United Kingdom (English) [30] was given to participants on days 7, 14 and 6 weeks. This self-administered questionnaire covers eight domains of both physical and mental health and has been used during the validation of other disease specific health measures across a wide range of conditions [31, 32]. The SF-36v2 Acute has a recall period of 1 week and therefore made it appropriate for use in evaluating the new OARS and OACS. Prior hypotheses for correlations were considered. These included that the highest correlations would be found between OARS domains and SF-36 v2 Acute dimensions between the following: OARS pain with the SF-36v2 Acute bodily pain; OARS nausea and feeling unwell with SF-36v2 Acute domain of general health; OARS fatigue and sleep with SF-36v2 Acute vitality; and OARS improving function and mobility with SF-36v2 Acute physical functioning.

#### Analysis

In phase four, validation and reliability was tested including scale generation and testing scale properties, descriptive statistics and frequency tables, internal consistency and construct validity. The SF-36, a previously validated generic health measure, was administered alongside the newly developed OARS and OACS to provide comparison and correlation for construct validity for the new measures. In addition, responsiveness and sensitivity to change were reported. The initial testing time point was measured and compared with the means of additional testing points through 6 weeks.

SPSS 25 software was used for analyses in phases three and four (IBM Corp. Released 2017. IBM SPSS Statistics for Windows, Version 25.0. Armonk, NY: IBM Corp.). Recommended scoring algorithms were utilised for the SF-36v2 (Quality Metric Health Outcomes™ Scoring Software 5.0; 2016).

#### Sample size calculations and considerations

In testing of psychometric properties, larger samples are often considered desirable [33]. Sample sizes based on five to ten times the number of respondents as items are often quoted in the literature [34, 35]. This guideline was used in the testing phases for both OARS and OACS.

## Results

### Study samples

Participant demographic and surgical characteristics for each of the study phases are presented in Table 1. The planning phase participants are presented in Table 2. Participants ranged in age from 20 to 92 years of age. These participants represented a range of ethnicities, duration of diseases and lower limb joint replacements. In the planning phase, these participants represented a range of healthcare careers, and years of experience caring for orthopaedic patients undergoing lower limb joint replacement.

**Table 1:** Participant characteristics

Stages	Stage One: Item generation and initial questionnaire development		Stage Two: Item reduction and scale generation. Testing reliability and validity	
Parameter/Study phase	Phase One	Phase Two	Phase Three	Phase Four
Age (years)	71.0 (10.3) 45–92	67 (11.3) 50–81	66.1 (9.9) 38–87	68 (10.9) 20–87
Sex Male/Female (% male)	14/16 (46.7)	16/18 (47)	71/97 (42)	65/90 (42)
Ethnicity	WB 28 (93.3)	WB 32 (94.1)	WB 157 (93.5)	WB 151 (97.4)
	OW 2 (6.7)	OW 2 (5.9)	OW 7 (4.2)	OW 1 (0.6)
			WI 2 (1.2)	I 1 (0.6)
			Af 1 (0.6)	Ar 1 (0.6)
			OA 1 (0.6)	C 1 (0.6)
Duration of disease (years)	4.6 (4.1) 0.67–20	4.4 (4.2) 0.5–30	6.2 (6.8) 0.5–43	6.2 (9.3) 0.5–74
Length of stay (days)	28.5 (24.0) 0–63	3.15 (2.83) 0–8	2.3 (2.5) 0–15	2.6 (2.7) 0–21
Type of surgery	THA 16 (53.3)	THA 9 (26)	THA 70 (41.7)	THA 58 (37.4)
	TKA 11 (36.7)	TKA 9 (26)	TKA 26 (15.5)	TKA 33 (21.3)
	UKA 2 (6.7)	UKA 12 (35)	UKA 54 (32.1)	UKA 55 (35.5)
	Rev. THA 1 (3.3)	Rev. THA 1 (3)	PFJ 2 (1.2)	Rev. THA 6 (3.9)
		Rev. TKA 3 (9)	Rev. THA 7 (4.2)	Rev. TKA 3 (1.9)
			Rev. UKA/TKA 9 (5.4)	
Employment	Employed 7 (23.3)	Employed 16 (47)	Employed 67 (39.9)	Employed 38 (24.5)
	Retired 21 (70)	Retired 18 (53)	Retired 91 (54.2)	Retired 110 (71)
	Disabled 2 (6.7)		Disabled 10 (5.9)	Disabled 7 (4.5)
Living situation	Alone 6 (20)	Alone 8 (24)	Alone 41 (24.4)	Alone 27 (17.4)
	Family 24 (80)	Family 26 (76)	Family 127 (75.6)	Family 128 (82.6)
Home situation	One level 4 (13.3)	One level 6 (18)	One level 37 (22)	One level 20 (12.9)
	Up and down stairs 26 (86.7)	Up and down stairs 28 (82)	Up and down stairs 131 (78)	Up and down stairs 135 (87.1)

Data are n (%) or mean (SD) range. Ethnicity: *WB* White British, *OW* Other White, *WI* White Irish, *Af* African, *OA* Other Asian, *I* Indian, *Ar* Arabic, *C* Caribbean. Ethnicity categories from Office for National Statistics (ONS), Census 2011. Type of surgery: *THA* Total Hip Arthroplasty, *TKA* Total Knee Arthroplasty, *UKA* Unicompartmental Knee Arthroplasty, *PFJ* Patellofemoral Joint Replacement, *Rev. THA* Revision Total Hip Arthroplasty, *Rev. UKA/ TKA* Revision Unicompartmental Knee Arthroplasty/Total Knee Arthroplasty, *Rev. TKA* Revision Total Knee Arthroplasty

**Table 2:** Planning phase participant characteristics

**Stages** **Parameter**	**Stage One: Item generation and initial questionnaire development**			
Sex Male/Female (% male)	10/12 (45.5)			
Ethnicity	Arab 1 (4.5)			
	White British 14 (63.6)			
	African 1 (4.5)			
	Indian 1 (4.5)			
	Other White 4 (18.2)			
	Irish 1 (4.5)			
Employment role	Registered Nurse 5 (22.7)			
	Orthopaedic Surgeon 5 (22.7)			
	Anaesthetist 5 (22.7)			
	Physical Therapist 5 (22.7)			
	Occupational Therapist 2 (9.1)			
Length of time in career (years)	16.4 (9.2) 1–35			
Sub Group	Number	Sex Male/Female	Ethnicity	Length of time in career (years)
Nurses	5	0/5	White British 4	1–24
			African 1	
Surgeons	5	5/0	Arab 1	8–21
			White British 3	
			Other White 1	
Anaesthetists	5	5/0	White British 2	15–22
			Indian 1	
			Other White 2	
Physiotherapists	5	0/5	White British 3	5–33
			Other White 2	
Occupational therapists	2	0/2	White British 2	12–35

Data are n (%) or mean (SD) range. Ethnicity categories from Office for National Statistics (ONS), Census 2011

### Conceptual framework

The development of a conceptual model or theoretical underpinning is essential when developing a new tool [12]. This framework was developed from the qualitative work in both the Planning Phase and Phase One interview analyses (Table 3). It provided a necessary framework around which the items and purpose of the questionnaire could be prepared. In the development of a PROM, or change measure, content validity is an essential component, as it ensures that the constructs of the topic, in these aspects of recovery from joint replacement surgery, are covered by the measure.

**Table 3:** Conceptual framework

Conceptual Framework	Theme	Subtheme
Recovery following lower limb arthroplasty	Pain	Swollen
		Pain at night
		Constant pain
		Feeling faint
		Feeling in general
		Comfort
		Joint pain
		Muscle spasms
		Pain killers
	Improving function and mobility	Difficulty getting into bed
		Not able to stand or walk
		Movement and mobility
		Going up and down stairs
		Strength
	Experiences of healthcare	Coping at home
		Appetite
		Mood
		Feeling sick or woozy
		Constipation
		Unwell
		Disorientated
	Fatigue and sleep	Feeling tired
		Not sleeping well
		Difficulty sleeping
		Not sleeping due to pain
		Energy level
		Feeling overall
	Support from others	Family
		Friends
		Worry
		Preoperative hip school
		After discharge
	Involvement in/understanding of care decisions	Knowing enough to make decisions
		Anaesthetic
		Giving you what they think you need
	Behaviour/ coping	Putting up with it
		Knowing my capabilities
		Listening to what you are being told
		Having faith in care providers
		Physical crutches
		Mental crutches

By utilising both patient data and an expert group of healthcare providers, this not only strengthened the robustness of the tool, but also ensured that it would make sense in the clinical setting in which it would be used. Whilst expert opinion aided the development of the semi-structured interview schedule, the items were generated solely on the basis of patient reports in Phase One of the study. Existing literature in the field was also consulted to reinforce the model.

### Stage one: item generation and initial questionnaire development

#### Planning phase

A total of 22 participants were included in the study (Table 2). All have been working in the care of the arthroplasty patient for an average of 16.4 years (SD = 9.2). All participants work in the same Orthopaedic hospital setting.

Three main themes evolved from the interviews and were mentioned by all interviewees: immediate recovery issues (*n* = 22), discharge criteria (*n* = 22), and priorities during hospitalisation from healthcare providers’ perspective (*n* = 22).

#### Phase one

Thirty patients receiving with hip or knee joint replacement surgery were recruited to phase one of study (Table 1). All participants were recruited in the same orthopaedic hospital. The duration of disease ranged from 0.67 years to 20 years (mean = 4.6; SD = 4.1).

Seven main themes evolved from the interviews and were discussed by all participants: ‘improving function and mobility’ (*n* = 30), ‘pain’ (*n* = 30), ‘effects of healthcare on outcomes’ (*n* = 30), ‘support from others’ (*n* = 30), ‘involvement and understanding of care decisions’ (*n* = 30), ‘behaviour and coping’ (*n* = 30) and ‘fatigue and sleeping’ (*n* = 30).

Phase one patient interviews provided statements for potential inclusion as items in the pilot questionnaires to be tested in phase two. Initially 136 statements were drafted for consideration. These draft statements were approved by the expert and patient panel to be evaluated in Phase Two. From this list of patient statements, between six and 12 were chosen by the panel for each thematic area. These covered recovery topics in full and were intended to be sensitive to changes following surgery. By discussion and exploration of the draft statements, it was determined that the items would be separated into two patient-reported questionnaires: one PROM and one change measure. Therefore the Oxford Arthroplasty Early Recovery Score (OARS) and Oxford Arthroplasty Early Change Score (OACS) were developed. Tables 4 and 5 show the pilot OARS and OACS items, themes and participant IDs. Healthcare professionals stated that these could be of great use in the clinical area. Patients had expressed interest in seeing how things had changed, and hopefully improved, since surgery.

**Table 4:** Pilot OARS items and themes breakdowns

Phase Two OARS Item	Theme	Participant ID
1. I have felt unwell	Effects of healthcare on outcomes	2, 11
2. I have felt disorientated	Effects of healthcare on outcomes	11, 18
3. I have felt tired	Fatigue and sleep	14
4. I have felt faint	Pain	18
5. It has been quite painful	Pain	3, 11, 13, 25
6. I have had constant pain	Pain	13
7. I have worse pain at night	Pain	13
8. It has felt swollen	Pain	10, 13, 26
9. I have difficulty getting into bed	Improving function and mobility	8
10. I am not able to stand	Improving function and mobility	11
11. I am not able to walk	Improving function and mobility	11
12. I have not slept well	Fatigue and sleep	18, 26
Which question 13a or 13b do you prefer? 13a. I have found it quite hard to sleep or 13b. I have found it difficult to sleep	Fatigue and sleep	6, 21
14. I have not been able to sleep due to the pain	Fatigue and sleep	30
15. I have felt sick	Effects of healthcare on outcomes	2, 4, 11, 13, 17, 18, 20, 21, 23, 24, 26
16. I have felt woozy	Effects of healthcare on outcomes	4
17. I have been constipated	Effects of healthcare on outcomes	13, 23, 24
18. I have lost my appetite	Effects of healthcare on outcomes	2, 24, 25

**Table 5:** Pilot OACS items and themes breakdowns

Phase Two OACS Item	Theme	Participant ID
1. Your ability to stand compared to prior to the operation?	Improving function and mobility	11
2. Your ability to move compared to prior to the operation?	Improving function and mobility	1, 3, 8, 16
3. Your movement compared to prior to the operation?	Improving function and mobility	7, 19, 30
4. Your mobility compared to prior to the operation?	Improving function and mobility	13
5. Your ability to walk compared to prior to the operation?	Improving function and mobility	3, 15, 16, 27
6. Your ability to go upstairs compared to prior to the operation?	Improving function and mobility	1, 5, 7, 17, 22, 27, 28
7. Your ability to go down stairs compared to prior to the operation?	Improving function and mobility	1, 5, 7, 17, 22, 27, 28
8. Your strength compared to prior to the operation?	Improving function and mobility	20, 30
9. Your pain compared to prior to the operation?	Pain	2, 5, 6, 9, 12, 13, 14, 15, 18, 19, 20, 21, 23, 24, 29, 30
10. How you feel today as compared to prior to the operation?	Pain	9, 10, 25, 27
11. How comfortable you feel today compared to prior to the operation?	Pain	7, 10, 30
12. Your pain when walking as compared to prior to the operation?	Pain	2, 5, 6, 8, 9, 12, 13, 14, 15, 17, 18, 19, 20, 21, 23, 24, 29, 30
13. Your pain in the joint today compared to prior to the operation?	Pain	18, 30
14. Your pain at night compared to prior to the operation?	Pain	20
15. Your pain when getting into bed compared to prior to the operation?	Pain	8
16. Your muscle spasms compared to prior to the operation?	Pain	20
17. The amount of pain killers you need to take as compared to prior to the operation?	Pain	29
18. Your ability to cope at home compared to prior to the operation?	Effects of healthcare on outcomes	21
19. Your appetite as compared to prior to the operation?	Effects of healthcare on outcomes	24, 25
20. Your mood compared to prior to the operation?	Effects of healthcare on outcomes	7, 18, 30
21. Your energy level compared to prior to the operation?	Fatigue and sleep	2
22. Your sleep as compared to prior to the operation?	Fatigue and sleep	2, 17, 21, 25, 26, 28, 30
23. How you feel overall compared to prior to the operation?	Fatigue and sleep	9, 11, 17, 19
24. Your pain when trying to sleep compared to prior to the operation?	Fatigue and sleep	17
25. Your ability to sleep through the night compared to prior to the operation?	Fatigue and sleep	30

The OARS and OACS evolved through multiple iterations prior to their completion for use in Phase Two. Careful consideration was given throughout to both the items themselves and to the answer response options. The wording rubric and instructions for completion also developed and improved through this process. Some themes were removed from the item selection pool because they related more to experiences than recovery and quality of life. Future work may incorporate an experiences questionnaire, but this was out with the scope of this study.

#### Phase two

A total of 34 participants were included in phase two of the study (Table 1). The duration of disease ranged from 0.5 years to 30 years (mean = 4.4; SD = 4.2). Overall patients liked the questions and layout of the OARS and OACS. Cognitive debrief interviews (*n* = 34) were used to assess items for face and content validity.

‘It was easy to fill in and they are all questions that are relevant’ (Participant 6).

When asked if the questions made sense or if any were confusing, patients again appeared to be happy with the content and wording.

‘They all made sense yeah’ (Participant 22).

‘They were dead easy and straight forward’ (Participant 30).

Patients were asked if there were any additional questions they would like to have been asked or if they felt anything had been omitted.

‘I think it covered most bases. The questions themselves were good questions, yes’ (Participant 8).

‘I might on reflection think of something else but at the moment I would have said that, that was fine’ (Participant 11).

‘I don’t think there was anything (missing). I think you pretty well covered everything. No I don’t think so’ (Participant 15).

Item 13 in the OARS (Table 4) contained two answer options as both had been expressed by patients. The answer options were 13a: I found it quite hard to sleep and 13b: I have found it difficult to sleep. Patients in phase two had a preference for 13b so that was selected for inclusion in the Phase Three OARS.

Following feedback from participants in phase two, four items were removed from the OACS:

- Item 3. Your movement compared to prior to the operation?-Seen as a duplicate to item 2: Ability to move compared to prior to the operation?
- Items 9. Your pain compared to prior to the operation? And Item 10. How you feel today as compared to prior to the operation?-Disliked by patients as seen as too similar to other questions
- Item 17. The amount of pain killers you need to take as compared to prior to the operation?-Too ambiguous per patients

Patients in the midst of an experience of immediate postoperative issues were able to elicit direct responses to the pilot items in the PROM and change measure during their hospital stay. These ideas were confirmed by the expert panel of healthcare professionals. The opinions from patients on the timing, wording of items and the answer options, provided essential feedback which shaped the next version of both the OARS and OACS questionnaires for testing and validation in the subsequent phases.

As patients were only administered the questionnaires once during this phase, they reported being keen to test the OARS and OACS at different time points and see if they would answer differently. This response was positive as the measures will be tested and validated over different time points during the recovery period in the next phases.

### Stage two: item reduction and scale generation. Testing reliability and validity

#### Phase three

Out of 168 participants, 161 returned their questionnaires at time point one (day 1 postoperatively) (missing *n* = 7, 4.2%). Before principle component analysis (PCA) was performed, KMO and Bartlett’s test of sphericity were performed to determine the suitability of the data for factor analysis. These appeared favourable for both the OARS and OACS with KMO .835 and .936 respectively and Bartlett’s test of significance .001. Consequently the data from both measures appeared amenable to factor analytic techniques.

### Oxford arthroplasty early recovery score (OARS)

EFA indicated that over 64% of the variance could be explained by four factors. Upon analysis of the data over 35% of the variance was explained by one factor. The eigenvalue for the first factor was 6.398. The second factor explained over 12% of the variance (eigenvalue 2.238). The remaining two factors explained 9.7% and 7% of the variance respectively. Both had eigenvalues over 1.

Inspection of the items suggested that the factors were tapping aspects of pain, sleep, nausea and feeling unwell, and mobility respectively (Table 6).

**Table 6:** Phase 3 18-item OARS day one item factor loadings

Rotated Component Matrix^**a**^				
	Component			
	1	2	3	4
OARS Item 6: I’ve had constant pain in the affected area	.779	.078	.257	.213
OARS Item 5: It’s been painful in the affected area	.774	.021	.248	.224
OARS Item 7: I’ve had pain at night in the affected area	.768	.071	.276	.189
OARS Item 8: The affected area has felt swollen	.665	.159	.002	.003
OARS Item 3: I’ve felt tired	.576	.337	.037	−.147
OARS Item 17: I’ve been constipated	.317	.263	.011	.042
OARS Item 16: I’ve felt woozy	.027	.878	.032	.105
OARS Item 15: I’ve felt sick	.086	.821	.123	.146
OARS Item 4: I’ve felt faint	.120	.755	.063	.057
OARS Item 1: I’ve felt unwell	.452	.614	.195	.114
OARS Item 2: I’ve felt disorientated	.274	.542	.027	.170
OARS Item 18: I’ve lost my appetite	.100	.540	.121	.339
OARS Item 13: I’ve found it difficult to sleep	.114	.080	.919	.137
OARS Item 12: I’ve not slept well	.141	.118	.911	.174
OARS Item 14: I’ve not been able to sleep due to the pain in the affected area	.404	.152	.739	.169
OARS Item 10: I’ve not been able to stand	.199	.178	.184	.857
OARS Item 11: I’ve not been able to walk	−.059	.289	.264	.804
OARS Item 9: I’ve had difficulty getting into bed	.462	.156	.056	.579
Extraction Method: Principal Component Analysis. Rotation Method: Varimax with Kaiser Normalization.^a^				

^a^Rotation converged in 6 iterations

Four items were removed due to low loadings and being disliked by patients/being ambiguous. Reanalysis following item reduction by EFA produced the same four factors resulting in similar variance (14 items) 69% and loadings.

The dimensions of the OARS questionnaire displayed good internal consistency and reliability with Cronbach’s alpha ranging between .74–.89 (Table 7). Item-to-total correlations are a measure of dimensionality and are also presented in Table 7.

**Table 7:** Phase three day 1 14 item OARS item to total correlations and internal reliability

Domain	Item	Corrected Item to Total Correlation	Cronbach’s Alpha
**Pain**			.74
	3. I’ve felt tired	.434	
	5. It’s been painful in the affected area	.647	
	7. I’ve had pain at night in the affected area	.629	
	8. The affected area has felt swollen	.459	
**Nausea and feeling unwell**			.78
	1. I’ve felt unwell	.586	
	4. I’ve felt faint	.561	
	15. I’ve felt sick	.695	
	18. I’ve lost my appetite	.499	
**Fatigue and sleep**			.89
	12. I’ve not slept well	.853	
	13. I’ve found it difficult to sleep	.829	
	14. I’ve not been able to sleep due to the pain in the affected area	.699	
**Increasing function and mobility**			.79
	9. I’ve had difficulty getting into bed	.501	
	10. I’ve not been able to stand	.776	
	11. I’ve not been able to walk	.659	

### Oxford arthroplasty early change score (OACS)

EFA indicated that over 70% of the variance could be explained by two factors. Upon analysis of the data over 63% of the variance was explained by one factor. The eigenvalue for the first factor was 13.207. The eigenvalue for the second factor was very low (1.592) and the amount of variance (7.6%) that was explained by the second factor was also low. Inspection of the items suggested that the two factors were primarily addressing three separate issues of mobility, pain, and sleep.

The second factor did not appear to be meaningful, and furthermore, the two separate factors did not appear to be meaningful on their own (Table 8). Consequently, it was decided to determine if a single forced-factor would make the most parsimonious solution [36]. Reanalysis of the remaining 14 items was performed following item reduction and similar variance and factor loadings were found.

**Table 8:** 21-item OACS day one item factor loadings

Rotated Component Matrix^**a**^		
	Component	
	1	2
OACS item 4: Your ability to walk compared to before the operation?	.888	.318
OACS item 3: Your mobility compared to before the operation?	.872	.354
OACS item 2: Your ability to move compared to before the operation?	.871	.320
OACS item 5: Your ability to walk up stairs compared to before the operation?	.865	.288
OACS item 6: Your ability to walk down stairs compared to before the operation?	.859	.284
OACS item 1: Your ability to stand compared to before the operation?	.841	.337
OACS item 9: Your pain in the affected area when walking as compared to before the operation?	.735	.387
OACS item 14: Your ability to cope at home compared to before the operation?	.684	.436
OACS item 10: Your pain in the affected area compared to before the operation?	.671	.518
OACS item 12: Your pain when getting into bed compared to before the operation?	.668	.482
OACS item 7: Your strength compared to before the operation?	.645	.368
OACS item 8: How comfortable you feel when sitting compared to before the operation?	.588	.514
OACS item 20: Your pain when trying to sleep compared to before the operation?	.437	.766
OACS item 18: Your sleep compared to before the operation?	.314	.755
OACS item 21: Your ability to sleep through the night compared to before the operation?	.325	.751
OACS item 19: How you feel overall compared to before the operation?	.476	.746
OACS item 16: Your mood compared to before the operation?	.292	.728
OACS item 13: Your muscle spasms in the affected area compared to before the operation?	.147	.701
OACS item 11: Your pain in the affected area at night compared to before the operation?	.452	.678
OACS item 15: Your appetite compared to before the operation?	.265	.629
OACS item 17: Your energy level compared to before the operation?	.535	.586
Extraction Method: Principal Component Analysis. Rotation Method: Varimax with Kaiser Normalization.^a^		

^a^Rotation converged in 6 iterations

The OACS questionnaire displayed good internal consistency and reliability with an overall Cronbach’s alpha of .95. Item-to-total correlations are an additional measure of test reliability and are also presented for the OACS in Table 9. These analyses resulted in the final OARS and OACS questionnaires, which now contain 14 items.

**Table 9:** Phase three day 1 14 item OACS item to total correlations and internal reliability

OACS Item	Corrected Item to Total Correlation	Cronbach’s Alpha
		.95
1. Your ability to stand compared to before the operation?	.778	
4. Your ability to walk compared to before the operation?	.849	
5. Your ability to walk up stairs compared to before the operation?	.829	
6. Your ability to walk down stairs compared to before the operation?	.819	
7. Your strength compared to before the operation?	.714	
8. How comfortable you feel when sitting compared to before the operation?	.742	
10. Your pain in the affected area compared to before the operation?	.801	
9. Your pain in the affected area when walking compared to before the operation?	.784	
15. Your appetite compared to before the operation?	.597	
16. Your mood compared to before the operation?	.676	
17. Your energy level compared to before the operation?	.769	
18. Your sleep compared to before the operation?	.661	
19. How you feel overall compared to before the operation?	.810	
20. Your pain when trying to sleep compared to before the operation?	.756	

#### Internal consistency

Internal consistency for both the OARS and OACS were good, with overall Cronbach’s alpha of 0.87 and 0.95 respectively. Cronbach’s Alpha reliability analysis for items loaded in each factor are presented (Tables 7 and 9).

#### Phase four

Out of the 158 approached, a total of 155 participants were included in the study: 90 women (58%) and 65 men (42%). Response rates were 78–91%, with the mean time to complete being approximately 6 min. Participants were again given both the OARS and OACS on days 1, 2, 3, 7, 14 and 6 weeks. In addition, they received SF-36 v2 Acute on days 7, 14 and 6 weeks.

#### Scale generation and testing scale properties

Both the overall 14-item OARS and domains of the OARS were scored on a scale of zero to 100, with zero being poor recovery and 100 being positive and indicative of a good recovery. The OACS change measure was also scored across a scoring range of 100, with minus 50 being much worse than before surgery, to 50, being much better than before surgery. Zero indicates no change.

Overall questionnaire scores for the OARS, OACS and four OARS domain scores: pain; nausea and feeling unwell; fatigue and sleep; and improving function and mobility, can be seen in Tables 10, 11 and 12 respectively.

**Table 10:** Mean overall scores for OARS testing time points

	OARS day 1	OARS day 2	OARS day 3	OARS day 7	OARS day 14	OARS 6 weeks
Mean	35.07	39.49	46.27	54.46	62.10	72.41
CI	31.47–38.67	35.96–43.03	43.36–49.17	51.66–57.27	59.13–65.07	69.32–75.49
N	141	146	143	138	134	126
SD	21.64	21.61	17.59	16.68	17.37	17.50
Range	98.21	94.64	91.07	91.07	80.36	80.36
Minimum	0.00	0.00	0.00	7.14	19.64	19.64
Maximum	98.21	94.64	91.07	98.21	100.00	100.00

*CI* 95% Confidence Interval, *N* Number, *SD* Standard deviation

**Table 11:** Mean overall scores for OACS testing time points

	OACS day 1	OACS day 2	OACS day 3	OACS day 7	OACS day 14	OACS 6 weeks
Mean	− 27.07	−22.42	−18.27	−13.81	−3.98	11.51
CI	−30.02--24.13	−25.45--19.39	−20.80--15.74	−16.64--10.97	−7.40--0.57	7.78–15.24
N	125	124	131	127	121	121
SD	16.64	17.04	14.63	16.14	19.00	20.74
Range	80.36	76.79	73.21	78.57	89.29	91.07
Minimum	−50.00	−50.00	−50.00	−48.21	− 44.64	−41.07
Maximum	30.36	26.79	23.21	30.36	44.64	50.00

*CI* 95% Confidence Interval, *N* Number, *SD* Standard deviation

**Table 12:** Mean domain scores for OARS

	Day 1	Day 2	Day 3	Day 7	Day 14	6 weeks
**Pain**						
Mean	25.04	27.32	30.99	35.09	42.55	55.18
N	149	148	145	140	135	128
SD	20.99	21.54	17.08	19.11	21.60	26.51
Range	100	93.75	87.5	93.75	100	93.75
Minimum	0	0	0	0	0	6.25
Maximum	100	93.75	87.5	93.75	100	100
**Nausea and feeling unwell**						
Mean	44.83	46.90	55.17	68.66	78.47	86.12
N	150	149	145	140	135	127
SD	27.46	25.29	22.97	21.03	20.21	16.42
Range	100	100	100	87.5	81.25	93.75
Minimum	0	0	0	12.5	18.75	6.25
Maximum	100	100	100	100	100	100
**Fatigue and sleep**						
Mean	32.72	39.43	46.35	50.00	50.18	63.16
N	150	149	146	141	136	126
SD	31.51	31.73	28.28	27.53	30.61	28.99
Range	100	100	100	100	100	100
Minimum	0	0	0	0	0	0
Maximum	100	100	100	100	100	100
**Improving Function and Mobility**						
Mean	37.38	46.32	54.71	66.43	78.67	85.48
N	142	147	145	140	134	128
SD	30.07	26.82	23.89	21.16	17.08	16.24
Range	100	100	100	100	75	66.67
Minimum	0	0	0	0	25	33.33
Maximum	100	100	100	100	100	100

*N* Number, *SD* Standard deviation

In the presence of missing data, scores were not imputed for these participants. As it is part of a validation study, it is considered best practice to not impute data when constructing and testing the measurement properties of a new instrument [29].

#### Internal consistency reliability

In this final phase of testing, the OARS questionnaire again displayed good internal consistency and reliability with Cronbach’s alpha ranging between .77–.91. Similarly, the OACS questionnaire displayed an overall Cronbach’s alpha of 0.93.

#### Responsiveness and sensitivity to change

The OACS questionnaire was designed to be a highly sensitive change measure and as such was utilised for the purpose of defining significant change in the OARS. The OACS change measure was scored from negative 50, indicating poor recovery, to positive 50, indication a good recovery. As this change measure is designed for and by the early recovery joint replacement population, the OACS measure is designed to assess change and, as it has multiple items, it may be more granular than a single transition question [37, 38]. In order to estimate the minimally important change in the OARS PROM score, the OACS change measure results were reviewed by the expert group. The group included a psychometrician with extensive experience and interest in PROMs, two consultant arthroplasty surgeons and a surgical researcher. It was determined that the change in score would be used to calculate the minimally important change (MIC). It was determined, as previously discussed, that a change of between five and fifteen points out of 100, would indicate ‘minimal change’, with between five and minus five as ‘no change’. OACS change scores for each patient were reviewed between each testing time point. Patients that reported a minimal change for the OACS at each change time point were then grouped together and mean change in the OARS scores calculated.

Change from ‘-5’ to ‘5’ was considered ‘no change’ and change of between ‘-15’to ‘-5’ and ‘5’ to ‘15’ were considered minimal change. Participant responses were categorised at each time point and means calculated. OARS scores were tested, both in aggregate (positive and negative change together, sign corrected), as discussed by Guyatt [39, 40], and positive change only.

OACS change scores for each patient were reviewed between each testing time point. Patients that reported a minimal change for the OACS at each change time point were then grouped together and mean change in the OARS scores calculated. Mean change on the overall OARS scores suggest that a minimal important change (MIC) of approximately 13 points is significant to patients and of possible clinical significance too (Tables 13 and 14).

**Table 13:** Mean overall ‘some change’ aggregate OARS scores

Some change aggregate	day 1–2	day 2–3	day 3–7	day 7–14	day 14–6 weeks
Mean	8.53	15.21	12.05	10.16	14.23
CI	7.44–9.62	11.63–18.79	10.14–13.97	8.18–12.15	10.73–17.74
N	36	33	56	52	33
SD	3.23	10.09	7.14	7.12	9.89
Range	12.50	33.93	26.79	26.79	41.07
Minimum	1.79	0.00	1.79	0.00	0.00
Maximum	14.29	33.93	28.57	26.79	41.07

*CI* 95% Confidence Interval, *N* Number, *SD* Standard deviation

**Table 14:** Mean overall ‘some positive change’ OARS scores

Some positive change	day 1–2	day 2–3	day 3–7	day 7–14	day 14–6 weeks
Mean	8.63	16.57	12.55	10.76	15.27
CI	7.36–9.90	12.33–20.81	10.12–14.97	8.36–13.16	11.44–19.10
N	24	25	38	40	29
SD	3.01	10.28	7.37	7.52	10.06
Range	8.93	33.93	26.78	26.79	41.07
Minimum	5.36	0.00	1.79	0.00	0.00
Maximum	14.29	33.93	28.57	26.79	41.07

*CI* 95% Confidence Interval, *N* Number, *SD* Standard deviation

#### Construct validity

All correlations and dimensions were considered during this process. Spearman rank-order correlation coefficients, or Spearman’s rho, were measured between the dimensions of the OARS and SF-36v2 Acute (*n* = 142 participants) (Table 15). Moderate to strong correlations were found between many of the previously hypothesised domains of the OARS and SF-36v2 Acute. These included correlations between OARS pain and SF-36v2 Acute bodily pain; OARS nausea and feeling unwell with SF-36v2 Acute domains of general health and vitality; OARS fatigue and sleep with SF-36v2 Acute vitality; and OARS improving function and mobility with SF-36v2 Acute physical functioning. These associations were seen between domains on all time points.

**Table 15:** Spearman’s rho correlations for domains of the OARS and SF-36v2 acute health domain scores at days 7, 14 and 6 weeks

			Physical Functioning	Role-Physical	Bodily Pain	General Health	Vitality	Social Functioning	Role- Emotional	Mental Health
**Correlations Day 7**										
Spearman’s rho	Pain	Correlation	.388	.285	.505	.407	.443	.349	.410	.453
	Nausea and feeling unwell	Correlation	.259	.262	.226	.367	.409	.255	.359	.402
	Fatigue and sleep	Correlation	.269	.355	.454	.249	.420	.368	.368	.358
	Improving function and mobility	Correlation	.413	.295	.369	.272	.271	.263	.207	.289
**Correlations Day 14**										
Spearman’s rho	Pain	Correlation	.426	.412	.556	.331	.497	.458	.460	.479
	Nausea and feeling unwell	Correlation	.287	.298	.398	.380	.444	.287	.379	.438
	Fatigue and sleep	Correlation	.278	.322	.477	.231	.376	.332	.311	.355
	Improving function and mobility	Correlation	.542	.335	.409	.439	.390	.310	.360	.409
**Correlations 6 weeks**										
Spearman’s rho	Pain	Correlation	.481	.454	.659	.318	.569	.490	.468	.497
	Nausea and feeling unwell	Correlation	.379	.400	.403	.387	.573	.369	.488	.499
	Fatigue and sleep	Correlation	.403	.432	.536	.262	.604	.406	.353	.422
	Improving function and mobility	Correlation	.486	.517	.537	.370	.437	.419	.413	.394

## Discussion

In order to be considered as a valid measure, it is important that certain quality criteria are met and reported [17, 41]. The OARS and OACS have been developed and assessed in accordance with these criteria [42].

The OARS and OACS are appropriate for the hip and knee replacement population. The items have been developed and created directly from patient interviews and their own words. These have been derived from the qualitative interviews and themes that were confirmed with patients following creation. The subject matter and domains of items within the questionnaires have also been confirmed with participants. Participants were generally positive in their assessment of the questionnaires and were, in most cases, willing to take part. They reported being keen to see how things had changed over time. Thus, reaffirming that which the measures seek to address.

Both the OARS and OACS received response rates of 78–91%. Patients reported it was not a burden with the mean time to complete being approximately 6 min. Participants stated that completing the questionnaires provided a welcome distraction from their surgical recovery which was an unexpected finding as the questionnaires primary focus is recovery and change since the time of surgery. Returning the questionnaires in pre-paid envelopes was reported as giving patients a reason to get up and go to the post box during the early recovery period. In addition, further work will include the measures being transferred onto digital electronic delivery methods [43]. This will aid ease of delivery of the measures and facilitate receipt and processing of returned data.

The scores derived from both the OARS and OACS have the potential to be meaningful in terms of the clinical picture of recovery for patients at the group level, and in relation to how the domain scores of the OARS correlates to the scales of a generic health measure (SF-36v2 Acute). The minimally important change (MIC) for the OARS has been estimated and believed to be around 13 points. More work is required to fully explore and define clinically important differences and meaningful change. In the future, results will be explored to refine and define changes at the level of the individual.

The precision of the OARS and OACS has been demonstrated in terms of robust psychometric testing including exploratory factor analysis and removal of items with floor/ceiling effects. Both the OARS and OACS have demonstrated precision in their ability to distinguish between groups.

Both measures display good reliability testing for internal consistency with Cronbach’s alpha of between 0.77 and 0.93. An alpha of over 0.8 is desirable and it is recommended that for measures being used at the level of the individual, a higher Cronbach’s alpha of 0.9 is recommended. Reproducibility, in terms of test re-test, is not feasible because of the very nature of the work within this area of research, as patients are experiencing a sustained period of constant change and, hopefully, improvement during these 6 weeks.

Further testing of these questionnaires at the level of the individual is planned, which could provide evidence for their use in routine clinical care and assessment. In addition, further work investigating methods and cut-points to interpret change scores that are meaningful to patients, and not just clinicians, is needed and is currently underway. The measures are available for paper and pen completion and through digital electronic delivery methods. This will aid ease of delivery of the measures and facilitate receipt and processing of returned data. These questionnaires will now be used in both observational studies and clinical trials. This work will enable the next stage of important work using the measure to define and assess an optimal-recovery protocol for lower limb joint replacement.

Further testing of these questionnaires at the level of the individual in patient pathways and clinical research could bring real results to patients, healthcare providers, hospital trusts and clinical commissioning groups. These questionnaires will now go on to be used in both research studies and clinical trials. The research completed in this paper will now enable the next stage of important work using these measures to define and assess of an optimal-recovery protocol for lower limb joint replacement.

Healthcare providers and hospital bodies must continue work to progress and optimise the patient recovery pathway. Many areas could still be developed and therefore continue to make marked improvements for patients. Research using validated measures provides the opportunity, in combination with already used PROMS and patient data, to continue to make strides in recovery, patient satisfaction and outcomes.

Routine use of the new PROM and change measure could facilitate benchmarking and audit within the clinical area. In addition, potential research uses for these tools could include studies to evaluate improving post-operative pain regimens.

Being able to identify exactly what components are being utilised and making a difference in the private versus NHS setting could greatly improve the patient populations. This, in turn, has the potential to positively affect outcomes for both the National Health Service (NHS) and greater patient populations.

The promising measurement properties of the OARS and OACS, their relevance to patients, clinicians and other stakeholders, make them the ideal measurements to be used in randomised controlled trials that assess the efficiency of different interventions in this increasing patient population.

## Conclusion

An early recovery tool, the Oxford Arthroplasty Early Recovery Score (OARS), and a change measure, the Oxford Arthroplasty Early Change Score (OACS), have been developed. The OARS and OACS have been tested and validated in accordance with FDA guidelines [9] and best practice. They have been validated for use in the first 6 weeks following UKA, TKA and THA. Initial psychometric testing has shown some positive results, validity and sensitivity to change.

## Data Availability

The datasets generated and/or analysed during the current study are not publicly available yet but are available from the corresponding author on reasonable request.

## Abbreviations

CED: Concept elaboration document
CI: Confidence interval
EFA: Exploratory factor analysis
ES: Effect size
FDA: Food and Drug Administration
IP: Inpatient
KMO: Kaiser-Meyer-Olkin Measure of Sampling Adequacy
LOS: Length of stay
MIC: Minimally important change
NHS: National Health Service
OA: Osteoarthritis
OACS: Oxford Arthroplasty Early Change Score
OARS: Oxford Arthroplasty Early Recovery Score
OHS: Oxford Hip Score
OKS: Oxford Knee Score
PCA: Principal component analysis
PROMs: Patient-reported outcome measures
Rev. THA: Revision total hip arthroplasty
Rev. TKA: Revision total knee arthroplasty
Rev. UKA: Revision unicompartmental knee arthroplasty
SDS: Same-Day Surgery Discharge
SF-36: Short-Form 36-question Quality of Life Questionnaire
SD: Standard deviation
SPSS: Statistical Package for the Social Sciences
THA: Total hip arthroplasty
TKA: Total knee arthroplasty
TA: Translatability assessment
UK: United Kingdom
US: United States of America
USA: United States of America
VAS: Visual Analogue Scale
WHO: World Health Organisation
